# Prognostic value of pulmonary hypertension with a nomogram in acute myocardial infarction patients with reduced left ventricular function

**DOI:** 10.3389/fcvm.2024.1368139

**Published:** 2024-04-22

**Authors:** Qian Liu, Changying Zhao, Peizhu Dang, Yongxin Li, Yang Yan

**Affiliations:** ^1^ Department of Cardiovascular Surgery, The First Affiliated Hospital of Xi’an Jiaotong University, Xi’an, China; ^2^ Department of Cardiovascular Medicine, The First Affiliated Hospital of Xi’an Jiaotong University, Xi’an, China

**Keywords:** acute myocardial infarction, pulmonary hypertension, reduced left ventricular function, prognosis, nomogram

## Abstract

**Background:**

Pulmonary hypertension (PH) is a common prognostic factor for acute myocardial infarction (AMI) and its impact may increase when combined with reduced left ventricular function.

**Methods:**

This retrospective cohort study enrolled AMI patients with reduced left ventricular function at the First Affiliated Hospital of Xi'an Jiaotong University from January 2018 to January 2022. Basing on the systolic pulmonary artery pressure assessed by echocardiogram, patients were assigned to the PH group and control group. Propensity score matching (PSM) in sex, age and Killip classification was used to match patients between two groups. The primary outcome was defined as 1-year mortality rate, which were obtained from medical records and phone calls.

**Results:**

After the PSM, a total of 504 patients were enrolled, with 252 in both groups. No significant difference of the adjusted factors was observed between the two groups. The 1-year mortality rate was significantly higher in the PH group compared with the control group (15.5% vs. 5.3%, *P *< 0.001). In the cox regression analysis, PH (HR: 2.068, 95% CI: 1.028–4.161, *P *= 0.042) was identified as an independent risk factor, alongside left ventricular ejection fraction (HR: 0.948; 95% CI: 0.919–0.979; *P *< 0.001), creatine kinase-MB isoenzymes (HR: 1.002; 95% CI: 1.000–1.003; *P *= 0.010) and pro-brain natriuretic peptide (HR: 1.000; 95% CI: 1.000–1.000; *P *= 0.018) for the 1-year mortality in AMI patients with reduced left ventricular function. A nomogram was established using the above factors to predict the 1-year mortality risks in these patients.

**Conclusion:**

AMI patients with reduced left ventricular function showed higher 1-year mortality rate when concomitant with PH. Four independent risk factors, including PH, were identified and used to establish a nomogram to predict the 1-year mortality risks in these patients.

**Clinical Trials.gov ID:**

NCT06186713.

## 1 Introduction

Acute myocardial infarction (AMI) is a cardiac emergency caused triggered by acute blockage of the coronary arteries and necrosis of the myocardium (1). It is a main cause of sudden cardiac death, marked by acute onset, high incidence and poor prognosis (2). One of its most prevalent complications is the reduced left ventricular function (3–6). Generally, the larger the infarct size, the greater the likelihood of reduced left ventricular function (7). Previous studies indicated that the left ventricular dysfunction significantly affects the prognosis of AMI, even in the thrombolytic era (8, 9). The incidence of sudden cardiac death in AMI patients with reduced left ventricular function after discharge was 10.0% at 2.1 years and 13.2% at 3.1 years (10). However, the majority of existing temporal trends data in real-world experiences are restricted to patients diagnosed with heart failure (HF), without specific emphasis on those exhibiting reduced left ventricular ejection fraction (LVEF) (5).

Pulmonary hypertension (PH), defined as mean PAP ≥20 mmHg at rest, is a pathological condition characterized by a sustained increase in pulmonary artery pressure, which results from common left heart diseases or lung diseases (11). Elevated pulmonary artery pressure puts strain on the right ventricle and may lead to right heart failure and even death in the advanced stages (12). It has been reported that PH can also exacerbate myocardial hypertrophy and myocardial infarction in AMI patients, leading to a poor prognosis (13). PH is also a frequent complication of left ventricular dysfunction, and is associated with a high mortality and incidence of heart failure when it complicates left ventricular dysfunction (14).

Overall, PH has a negative effect on AMI patients and appears to be closely associated with reduced left ventricular function. However, its impact on AMI patients with reduced left ventricular function remains unclear. This retrospective study included AMI patients with reduced left ventricular function to investigate the prognostic value of PH in this specific type of patient. Meanwhile, a nomogram would be established basing on the identified independent risk factors, hoping to provide a novel risk stratification for them.

## 2 Methods

### 2.1 Study design and participants

**Figure 1:**
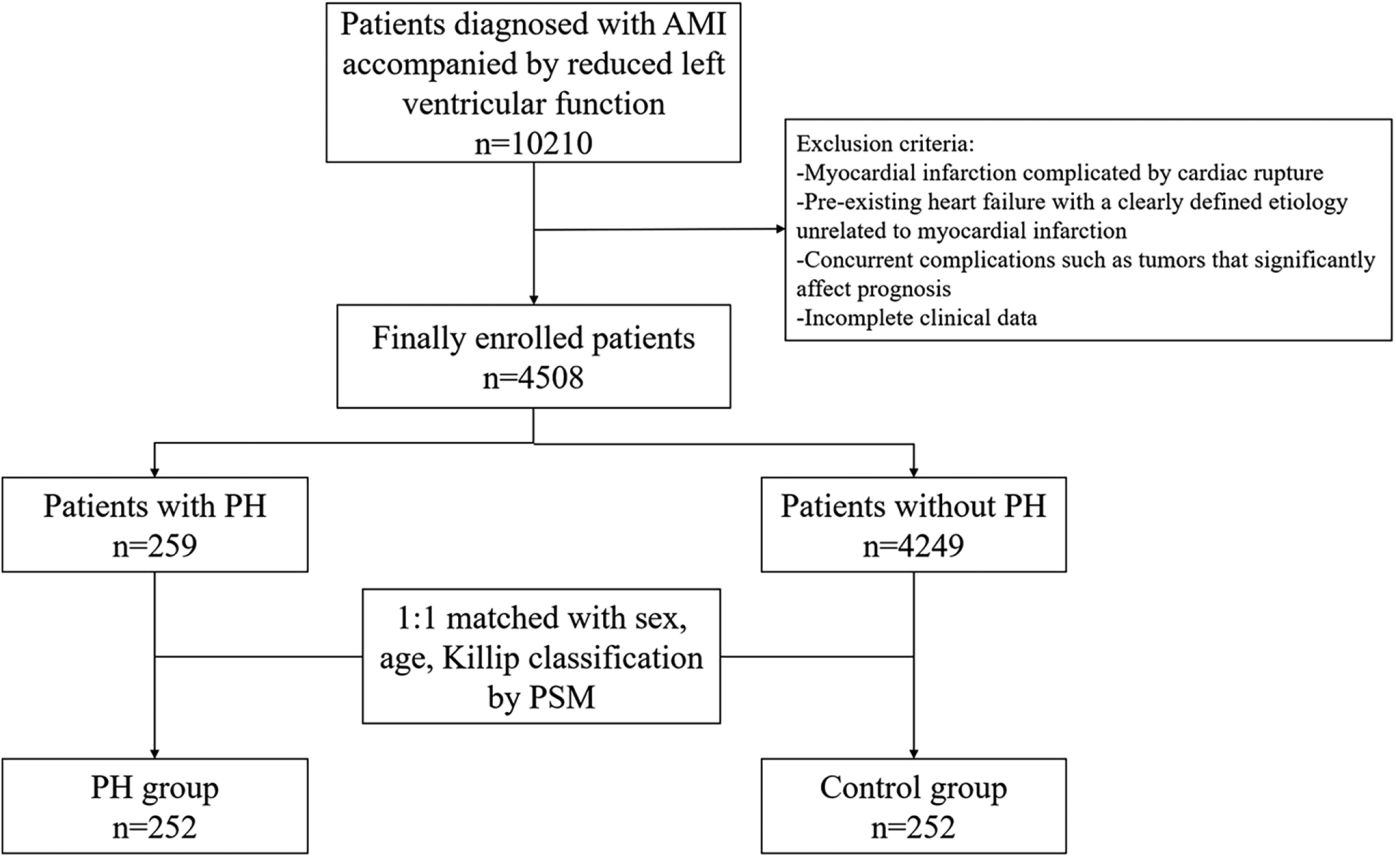
The flowchart of the study.

This retrospective cohort study included patients who were hospitalized for AMI with reduced left ventricular function at The First Affiliated Hospital of Jiaotong University from January 2018 to January 2022. The diagnosis of AMI was based on the universal definition criteria of the American College of Cardiology (15). Reduced left ventricular function was defined as LVEF less than 62% in men and 64% in women, as measured by echocardiography (16). The exclusion criteria were as follows: (1) AMI complicated by myocardial rupture; (2) Significant pre-existing heart failure; (3) Other comorbidities such as malignancy that may significantly affect prognosis; (4) Incomplete clinical data. The study flowchart was shown in Figure 1.

This study was approved by the Ethics Committee of the First Affiliated Hospital of Xi'an Jiaotong University (No. XJTU1AF2023LSK-406) and was conducted in accordance with the Declaration of Helsinki.

### 2.2 Grouping

The gold standard for the diagnosis of PH is the right heart catheterization examination. Nonetheless, the invasive nature of this examination makes it difficult to use as a routine test for AMI patients. As one of the optimal approaches for assessing potential PH, echocardiography was employed in this study (12).

Patients were categorized into the PH and control groups based on pulmonary artery pressure measured by echocardiography upon admission, which could reflect their baseline situation before treatments. The PH group comprised of patients with systolic pulmonary artery pressure (sPAP) ≥35 mmHg while the control group included those with sPAP <35 mmHg (17).

### 2.3 Study outcome and data collection

The primary outcome of this study was the 1-year mortality rate. Anonymized clinical data were collected from the Biobank of The First Affiliated Hospital of Xi'an Jiaotong University. The demographics characteristics, biochemical indicators, imaging results and clinical information of all patients were collected for subsequent analysis.

The follow-up period extended from discharge to either the occurrence of the primary outcome or one year following discharge. The survival status of patients was obtained via electronic medical records or communication with patients and their relatives through telephone conversations.

### 2.4 Propensity score matching

A 1:1 ratio of propensity score matching (PSM) was employed with a caliper value of 0.002 to minimize the effect of potential confounding factors, including gender, age, and Killip classification, and to establish an even distribution of selected variables across the two groups.

### 2.5 Statistical analysis

Continuous variables that conformed to a normal distribution were presented using the mean and standard deviation, and were compared using the Student's *t*-test. For continuous variables that did not conform to a normal distribution, the median with interquartile range (25th and 75th percentile) was presented and were compared using a Mann-Whitney *U*-test. Categorical variables were described as counts with percentages (*n*, %), and comparison was made using chi-square test or Fisher's exact test.

One-year mortality were compared using the Kaplan-Meier method and log-rank tests. Univariate and multivariate COX regression analyses were conducted to identify the independent risk factors. Variables with a *P*-value <0.10 in the univariate Cox regression would be included in the multivariate Cox regression analyses. The hazard ratio (HR) and 95% confidence intervals (CI) were calculated to determine the intensity of risk factors. The independent risk factors identified in the multivariate Cox regression were used to build a nomogram which could predict the 1-year mortality risks. The accuracy of the nomogram was evaluated by area under the curve (AUC), the consistency was assessed by the calibration curve, and the clinical values were tested by the decision curve analysis.

The SPSS 27.0 (SPSS Inc., USA) and R 3.1.2 (R Foundation for Statistical Computing, Austria) were used for statistical analysis. The main R software packages used for analysis were “survival” and “survminer”. A two-sided *P*-value <0.05 was considered statistically significant.

## 3 Results

A total of 4,784 AMI patients with reduced left ventricular function were included in this study. Among them, 259 (5.7%) patients were assigned to the PH group, and the rest were assigned to the control group. After PSM, there were 252 patients in both the PH and control groups.

### 3.1 PSM results

The sex, age and Killip classification for two groups were shown in Table 1. Before PSM, the PH group exhibited a lower percentage of male patients (70.3% vs. 81.8, *P* < 0.001) and a higher number of patients with Killip classification ≥3 (27.0% vs. 6.8%, *P* < 0.001). Additionally, the PH group had older ages [68.00 (60.00, 73.00) vs. 60.00 (52.00, 68.00), *P* < 0.001] compared to the control group. After the PSM, there was no significant difference in the aforementioned three variables between the two groups.

**Table 1:** Clinical characteristics of PH in AMI patients with reduced left ventricular function before and after PSM.

Characteristics	Before PSM (*N *= 4,508)			After PSM (*N* = 504)		
	PH group	Control group	*P*-value	PH group	Control group	*P*-value
	(*N* = 259)	(*N* = 4,249)		(*N* = 252)	(*N* = 252)	
Male (*n*, %)	182 (70.3)	3,477 (81.8)	<0.001	180 (71.4)	181 (71.8)	0.921
Age (years)	68.00 (60.00, 73.00)	60.00 (52.00, 68.00)	<0.001	68.50 (61.25, 74.00)	68.50 (61.25, 74.00)	0.317
Killip ≥3 (*n*, %)	70 (27.0)	290 (6.8)	<0.001	63 (25.0)	63 (25.0)	1.000

PH, pulmonary hypertension; AMI, acute myocardial infarction; PSM, propensity score matching.

### 3.2 Baseline characteristics

The demographic characteristics, biochemical examination and imaging results between two groups were presented in Table 2. Compared with the control group, PH group demonstrated lower levels of triglycerides [1.02 (0.76, 1.33) vs. 1.19 (0.83, 1.65), *P *< 0.001], total cholesterol [3.69 (3.05, 4.36) vs. 4.13 (3.30, 4.90), *P *< 0.001], creatine kinase-MB isoenzymes (CK-MB) [29.50 (15.00, 97.98) vs. 48.00 (18.00, 160.18), *P *= 0.008] and LVEF [4.13 (3.30, 4.90) vs. 47 (41, 55), *P *< 0.001]. Additionally, more patients in PH group were treated by calcium channel blockers (4.0% vs. 9.1%, *P *= 0.019). By contrast, the blood urea nitrogen [6.30 (5.02, 8.45) vs. 6.07 (4.89, 7.47), *P *= 0.011] and pro-brain natriuretic peptides [3,154.00 (1,310.75, 6,608.00) vs. 1,325.00 (447.28, 2,673.00), *P *< 0.001] were higher in the PH group, with longer in-hospital stay (5.85 vs. 4.25, *P *< 0.001) and more anemia patients (20.2% vs. 11.5%, *P *= 0.007).

**Table 2:** Differences in demographic and clinical characteristics of AMI patients with reduced left ventricular function with and without PH.

Characteristics	Total	PH group	Control group	*P*-value
	(*N* = 504)	(*N* = 252)	(*N* = 252)	
Smoker (*n*, %)				
Current smoking	58 (11.5)	22 (8.7)	36 (14.3)	0.051
Previous smoking	25 (5.0)	11 (4.4)	14 (5.6)	0.538
Drinker (*n*, %)	35 (6.9)	18 (7.1)	17 (6.7)	0.861
Diagnosis				
Non-STEMI	179 (35.5)	82 (32.5)	97 (38.5)	0.163
Anterior wall	118 (23.4)	60 (23.8)	58 (23.0)	0.833
Extensive anterior wall	62 (12.3)	33 (13.1)	29 (11.5)	0.588
Inferior wall	129 (25.6)	70 (27.8)	59 (23.4)	0.262
Posterior wall	52 (10.3)	26 (10.3)	26 (10.3)	>0.999
Others	16 (3.2)	10 (4.0)	6 (2.4)	0.310
Blood biochemistry				
D-dimer (mg/L)	0.75 (0.40, 1.47)	0.83 (0.50, 1.55)	0.64 (0.35, 1.42)	0.056
NEUT# (10^9^/L)	7.32 (5.29, 9.75)	7.17 (5.23, 9.84)	7.36 (5.29, 9.69)	0.861
LYMPH# (10^9^/L)	1.20 (0.88, 1.66)	1.19 (0.87, 1.68)	1.22 (0.91, 1.66)	0.430
WBC (10^9^/L)	9.25 (7.23, 11.82)	9.26 (7.25, 11.79)	9.25 (7.18, 11.83)	0.952
TG (mmol/L)	1.11 (0.77, 1.48)	1.02 (0.76, 1.33)	1.19 (0.83, 1.65)	<0.001
TC (mmol/L)	3.83 (3.17, 4.58)	3.69 (3.05, 4.36)	4.13 (3.30, 4.90)	<0.001
LDH (U/L)	331.00 (253.00, 581.75)	335.50 (254.25, 641.50)	328.00 (249.75, 523.00)	0.163
CK (U/L)	1,095.75 (335.00, 3,019.00)	277.50 (110.75, 887.00)	430.00 (131.00, 1,271.25)	0.066
CK-MB (U/L)	36.70 (16.00, 132.75)	29.50 (15.00, 97.98)	48.00 (18.00, 160.18)	0.008
Pro-BNP (pg/ml)	1,962.50 (651.35, 4,961.50)	3,154.00 (1,310.75, 6,608.00)	1,325.00 (447.28, 2,673.00)	<0.001
BUN (mmol/L)	6.16 (4.94, 7.90)	6.30 (5.02, 8.45)	6.07 (4.89, 7.47)	0.011
Cr (μmol/L)	66.00 (54.00, 86.00)	68.00 (56.00, 86.00)	65.00 (51.25, 86.75)	0.127
eGFR (ml/min/1.73 m^2^)	86.65 (69.03, 96.66)	88.90 (73.96, 96.81)	84.53 (64.81, 96.11)	0.096
Lactic acid (mmol/L)	1.9 (1.4, 2.9)	1.9 (1.3, 3.0)	2.0 (1.5, 2.9)	0.307
PaO_2_ (mmHg)	79.2 (66.5, 94.7)	77.9 (63.4, 91.1)	80.8 (68.1, 96.5)	0.116
Comorbidities (*n*, %)				
Hypertension	279 (55.4)	133 (52.8)	146 (57.9)	0.244
Diabetes	159 (31.5)	85 (33.7)	74 (29.4)	0.292
COPD	7 (1.4)	6 (2.4)	1 (0.4)	0.128
CKD	14 (2.8)	10 (4.0)	4 (1.6)	0.104
PVD	23 (4.6)	13 (5.2)	10 (4.0)	0.522
Stroke	68 (13.5)	36 (14.3)	32 (12.7)	0.602
Anemia	80 (15.9)	51 (20.2)	29 (11.5)	0.007
Medications (*n*, %)				
ACEI	13 (2.6)	6 (2.4)	7 (2.8)	0.779
β-blocker	5 (1.0)	4 (1.6)	1 (0.4)	0.369
ARB	11 (2.2)	6 (2.4)	5 (2.0)	0.760
CCB	233 (46.2)	10 (4.0)	23 (9.1)	0.019
Diuretic	6 (1.2)	3 (1.2)	3 (1.2)	1.000
Echocardiography				
LVEF (%)	45 (38, 52)	42 (37, 48)	47 (41, 55)	<0.001
Mitral regurgitation (%)	370 (73.4)	184 (73.0)	186 (73.8)	0.840
RV longitudinal diameter (mm)	17 (15, 19)	16 (15, 20)	17 (15, 18)	0.639
RV transverse diameter (mm)	28 (25, 30)	28 (26, 30)	27 (25, 30)	0.541
In-hospital stay (days)	4.94 (2.96, 7.59)	5.85 (3.59, 8.82)	4.25 (2.63, 6.63)	<0.001

AMI, acute myocardial infarction; PH, pulmonary hypertension; STEMI, ST-segment elevation myocardial infarction; NEUT#, neutrophil count; LYMPH#, lymphocyte count; WBC, white blood cell; TG, triglycerides; TC, total cholesterol; LDH, lactate dehydrogenase; CK, creatine kinase; CK-MB, creatine kinase-MB isoenzymes; Pro-BNP, pro-brain natriuretic peptide; BUN, blood urea nitrogen; Cr, creatinine; eGFR, estimated glomerular filtration rate; COPD, chronic obstructive pulmonary disease; CKD, chronic kidney disease; PVD, peripheral vascular disease; ACEI, angiotensin-converting enzyme inhibitors; ARB, angiotensin receptor blockers; CCB, calcium channel blockers; LVEF, left ventricular ejection fraction; RV, right ventricular.

### 3.3 Risk factors for 1-year mortality

**Figure 2:**
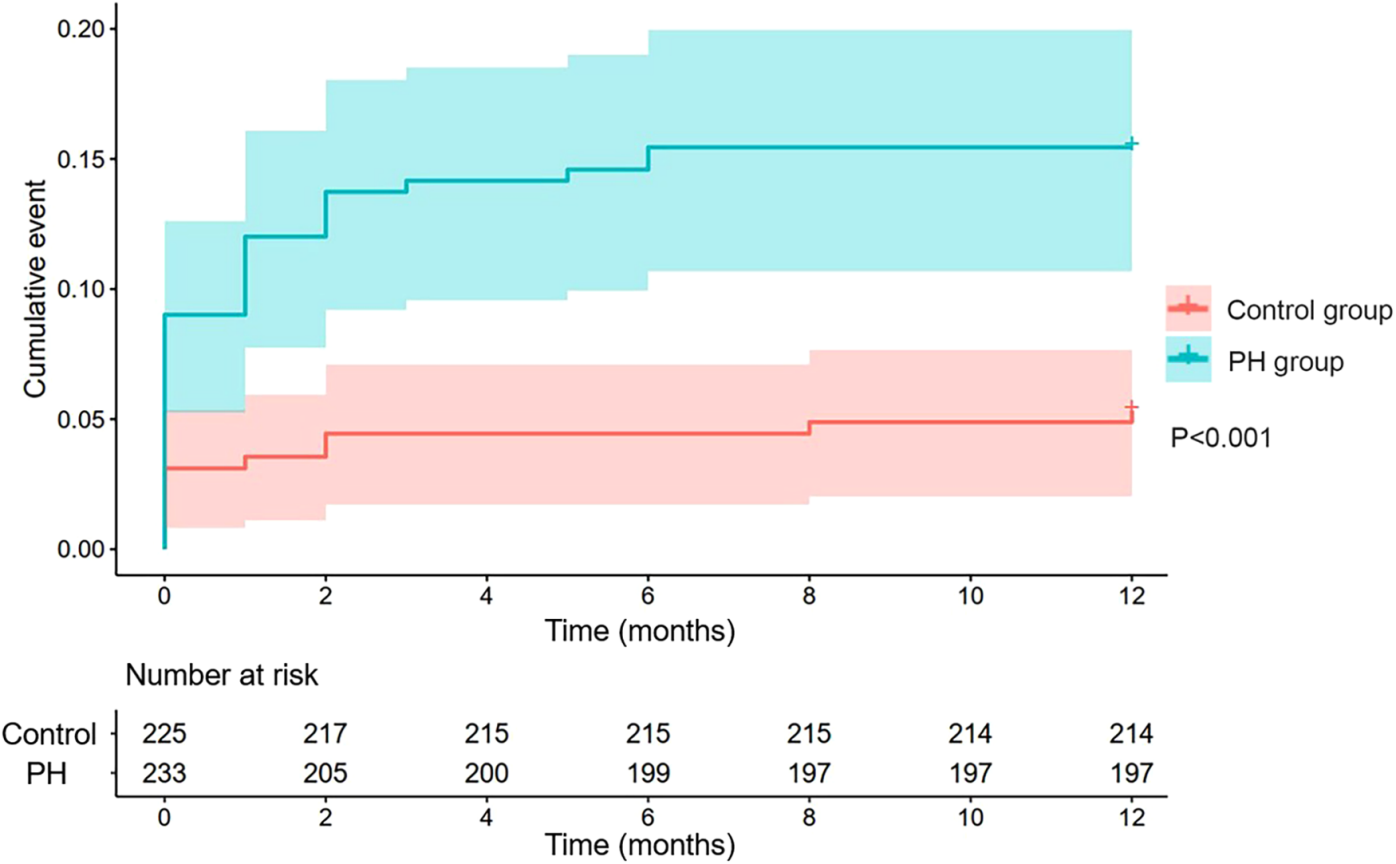
Kaplan-Meier curves of 1-year mortality rates in the PH group and control group (*P *< 0.001). PH, pulmonary hypertension.

A total of 46 (9.1%) patients were not available for follow-up, with 19 belonging to the PH group. Among others, 48 (10.48%) patients died during the follow-up time, of which 36 were in the PH group. The 1-year mortality rate was significantly higher in PH group than that in control group (15.5% vs. 5.3%, *P *< 0.001, Figure 2). The results of multivariate COX regression indicated that PH was an independent risk factor for 1-year mortality in AMI patients with reduced left ventricular function (HR: 2.068, 95% CI: 1.028–4.161, *P *= 0.042). Furthermore, LVEF (HR: 0.948; 95% CI: 0.919–0.979; *P *< 0.001), CK-MB (HR: 1.002; 95% CI: 1.000–1.003; *P *= 0.010) and Pro-BNP (HR: 1.000; 95% CI: 1.000–1.000; *P *= 0.018) were also found to be independent risk factors for poor prognosis in these patients (Table 3).

**Table 3:** Factors affecting outcomes in AMI patients with reduced left ventricular function.

Characteristics	Univariate model		Multivariate model	
	HR (95% CI)	*P*-value	HR (95% CI)	*P*-value
PH (%)	3.005 (1.563, 5.775)	<0.001	2.068 (1.028, 4.161)	0.042
Current smoking (%)	1.284 (0.576, 2.862)	0.541		
Previous smoking (%)	1.454 (0.452, 4.677)	0.531		
Drink (%)	0.919 (0.286, 2.957)	0.887		
TG (mmol/L)	0.848 (0.643, 1.117)	0.241		
TC (mmol/L)	0.539 (0.289, 1.004)	0.051	0.970 (0.717, 1.313)	0.844
CK-MB (U/L)	1.001 (1.000, 1.003)	0.070	1.002 (1.000, 1.003)	0.010
Hypertension (%)	0.619 (0.350, 1.095)	0.099	0.589 (0.330, 1.052)	0.074
COPD (%)	5.809 (1.802, 18.723)	0.003	2.901 (0.856, 9.834)	0.087
CKD (%)	2.506 (0.779,8.067)	0.123		
Anemia (%)	1.436 (0.716, 2.883)	0.308		
CCB (%)	0.045 (0.000, 8.473)	0.246		
Pro-BNP (pg/ml)	1.000 (1.000, 1.000)	<0.001	1.000 (1.000, 1.000)	0.018
LVEF (%)	0.933 (0.905, 0.961)	<0.001	0.948 (0.919, 0.979)	0.001

Abbreviations as in Tables 1, 2.

### 3.4 Nomogram to predict the 1-year mortality risk

**Figure 3:**
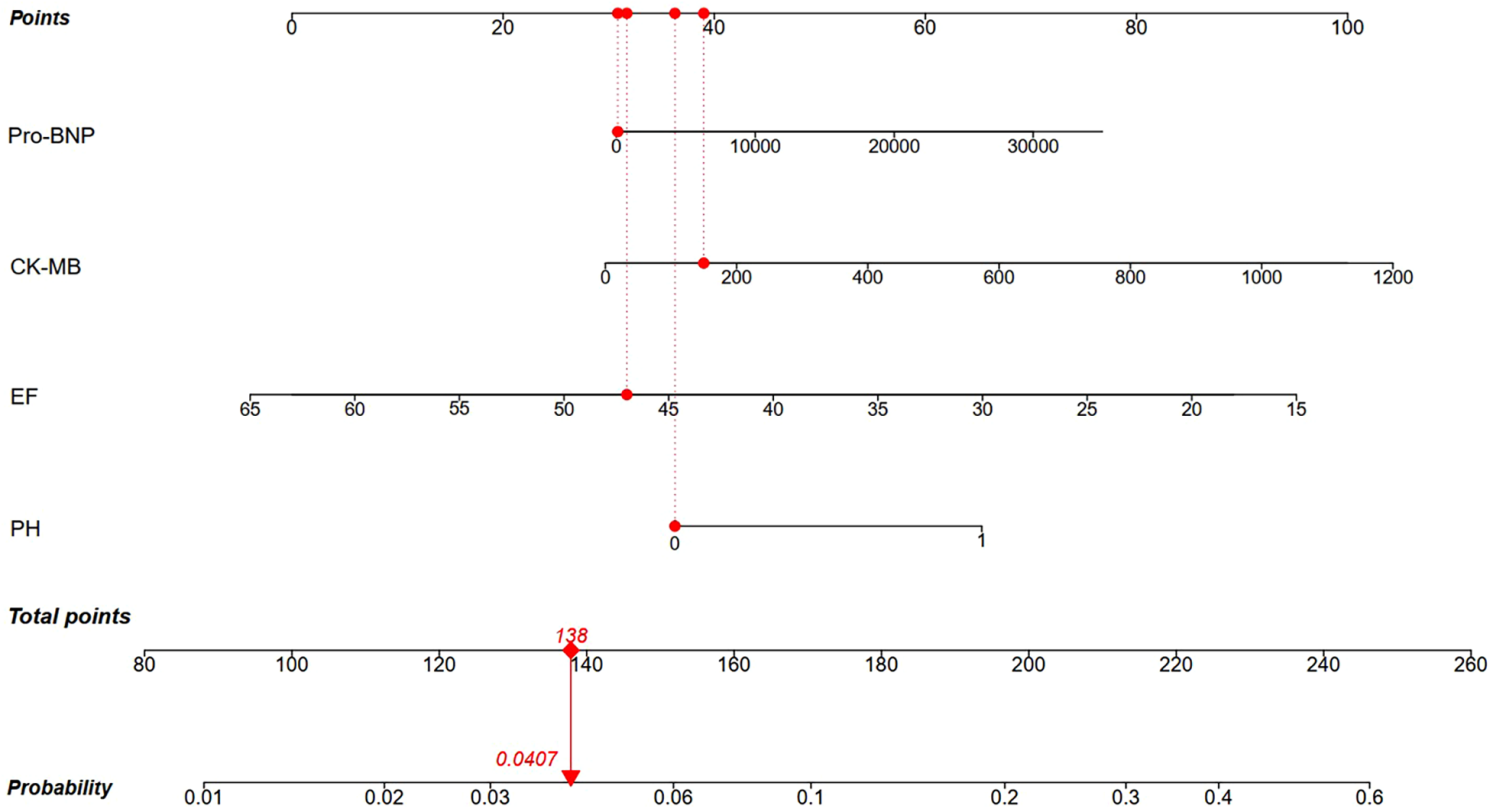
Nomogram for 1-year mortality risks in AMI patients with reduced left ventricular function basing on the four independent risk factors. Red line indicated a patient with a total point of 138 and 4.07% probability of 1-year mortality. Pro-BNP, pro-brain natriuretic peptide; CK-MB, creatine kinase-MB isoenzymes; LVEF, left ventricular ejection fraction; PH, pulmonary hypertension.

**Figure 4:**
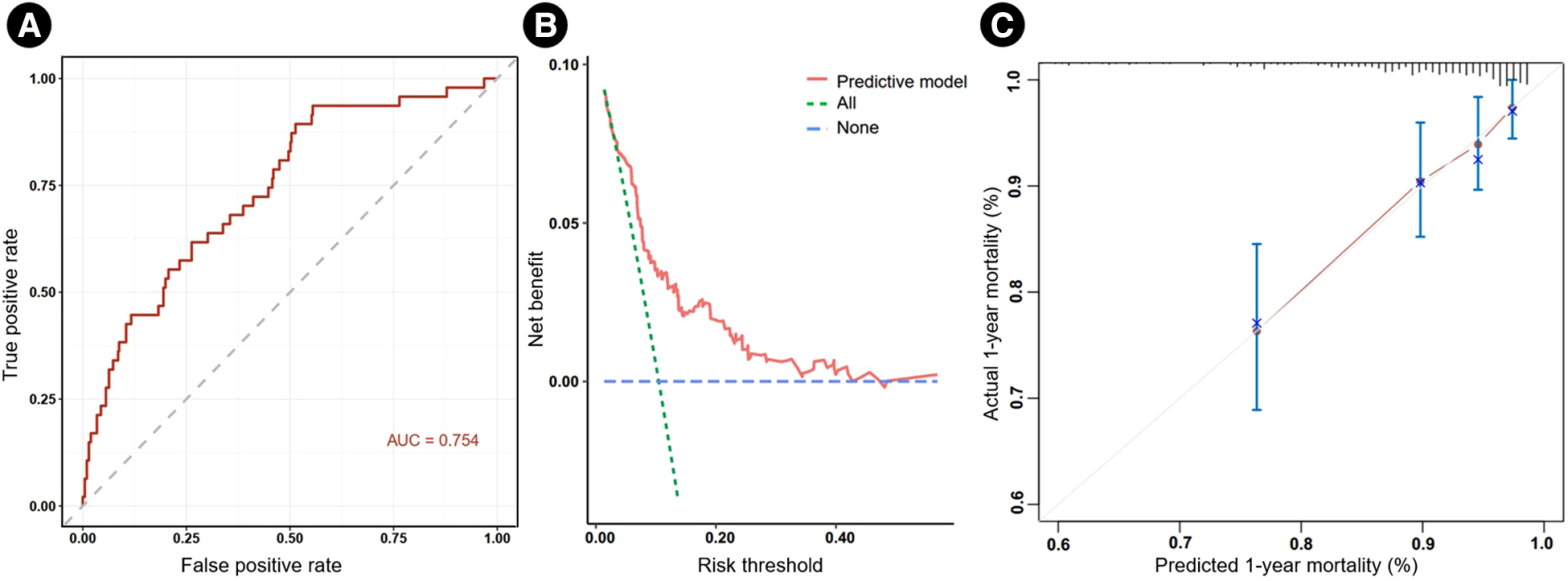
The receiver operating characteristic curve (**A**), calibration curve (**B**) and decision curve (**C**) of the nomogram. AUC, areas under the curve.

A nomogram was built using the four independent risk factors, PH, LVEF, CK-MB and Pro-BNP, in AMI patients with reduced left ventricular function (Figure 3). Patients could take a single point for each independent risk factor at admission. The four single point could be summed for a total point, which was related to a probability of 1-year mortality. The AUC value of this nomogram was 0.754. Meanwhile, at the total mortality rate (10.48%), the calibration curve was above the both “All” and “None” line. The decision curve also indicated good accuracy of the predictive model (Figure 4).

## 4 Discussion

In this retrospective cohort study, a total of 504 AMI patients with reduced left ventricular function were enrolled after PSM. At follow-up, the 1-year mortality rate was significantly higher in the PH group than that in the control group (15.5% vs. 5.3%, *P *< 0.001). PH could be considered as an independent risk factor, together with LVEF and CK-MB on admission, for 1-year mortality in AMI patients with reduced left ventricular function.

Despite the recent decrease in morbidity and mortality rates, AMI still poses a significant global health burden. Thorsten et al. demonstrated the impacts of infarct size and transmural extension of necrosis on the progressive remodeling of the left ventricle. These two factors could cause alterations in the structure and mechanical properties of the left ventricle myocardium, resulting in decreased diastolic and systolic function of the left ventricle (18). Meanwhile, pressure and volume overload after AMI could also lead to increased ventricular wall stress and impaired left ventricular function through a variety of mechanisms, including inflammation, energy metabolism and neuro-hormonal activation (19). Eventually, AMI patients with reduced left ventricular function always exhibit greater amounts of scarred or non-viable myocardium, leading to negative outcomes (7).

Current studies suggest that the global prevalence of PH is ≥1% and all age groups can be affected. PH is associated with poor prognosis in many diseases. The relationship between left heart disease and PH has been extensively reported in the literature, which accounts for 65%–80% of all cases (11). Furthermore, although right heart disease has received relatively little attention, a previous study suggested that PH may be a strong risk factor for both mortality and heart failure in AMI patients with impaired right ventricular function (20). It may be the reason that PH can trigger the progression of pulmonary vascular remodeling and the gradual increase in pulmonary arterial pressure, both of which can alter geometry and function of the right ventricle (21). Meanwhile, the increase in afterload triggers a series of mechanical and biochemical changes that may ultimately contribute to right heart failure. This PH-induced right heart failure will be more devastating for patients who already have reduced left ventricular function.

There have been studies that explored the role of prognostic role of PH in AMI patients with reduced left ventricular function. Barywani et al. enrolled AMI patients aged over 80 years with sPAP ≥40 mmHg, and found that elevated sPAP was an independent risk factor for one- and five-year all-cause mortality post-AMI in very elderly patients, which appeared to be a better prognostic predictor than LVEF (22). This conclusion is essentially consistent with this study, which applies to AMI patients across a broader age range. Meanwhile, reduced LV function is a common complication post AMI, and the focus of this study was on this specific type of patient. This study also identified LVEF, CK-BM and Pro-BNP at admission as independent risk factors for 1-year mortality. Basing on these four factors, a nomogram was built to predict the 1-year mortality risks for AMI patients with reduced left ventricular function, in which PH played an important role. Currently, there is no single independent factor that can adequately assess the prognosis of patients with AMI. This nomogram, combining these four factors, could be used as a quantitative risk stratification in clinical practice and alert physicians to pay special attention to patients with higher death risk.

In conclusion, PH is a progressive disease that often leads to a negative outcome. Although the cardiac catheterization provides the formal diagnosis, transthoracic echocardiography also plays a vital role in identifying high-risk patients. It is important to note sPAP measured by echocardiography and more attention should be paid to AMI patients with reduced left ventricular function who also have suspected PH on admission.

There are some limitations in this study. Firstly, it is a single-center retrospective study, limiting the credibility of this study. Secondly, while echocardiography has utility in assessing PH, it is insufficient to provide as precise an evaluation as the right heart catheterization. Finally, due to the limited patients, we did not conduct internal and external verification of the nomogram, which may introduce bias.

## 5 Conclusion

This retrospective cohort study found that PH, LVEF, CK-MB and Pro-BNP at admission may be the independent risk factors for 1-year mortality in AMI patients with reduced left ventricular function. A nomogram basing on these four independent risk factors could be used to predict the 1-year mortality risks for stratification. Particular attention should be given to AMI patients with reduced left ventricular function who were also suspect with possible PH by echocardiography.

## Data Availability

The raw data supporting the conclusions of this article will be made available by the authors, without undue reservation.
